# Clinical presentation and antimicrobial resistance of invasive *Escherichia coli* disease in hospitalized older adults: a prospective multinational observational study

**DOI:** 10.1007/s15010-023-02163-z

**Published:** 2024-01-25

**Authors:** Joachim Doua, Jesús Rodríguez-Baño, Rachel Froget, Padma Puranam, Oscar Go, Jeroen Geurtsen, Sanne van Rooij, Tuba Vilken, Inage Minoru, Izumi Yasumori, Bart Spiessens, Evelina Tacconelli, Lena M. Biehl, Joshua T. Thaden, Michal Sarnecki, Herman Goossens, Jan Poolman, Marc Bonten, Miquel Ekkelenkamp, on behalf of the COMBACTE-NET Consortium/EXPECT Study Group, Madison Violette, Sonal Munshi, Moussa Aitabi, Christine Lammens, Sofie Van Mieghem, Sandra Van Puyvelde, Basil Britto Xavier, Anna Maria Azzini, Elda Righi, Nicola Duccio Salerno, Giuliana Lo, Cascio Eleonora Cremonini, Álvaro Pascual, Reinaldo Espíndola, Virginia Palomo, Sarah Walker, Felicia Ruffin, Michael Dagher, Andreja Varjačić

**Affiliations:** 1 Janssen Research and Development, Infectious Diseases and Vaccines, Janssen Pharmaceutica, Beerse, Belgium; 2 Unidad Clínica de Enfermedades Infecciosas y Microbiología, Hospital Universitario Virgen Macarena, Seville, Spain; 3 Department of Medicine, University of Sevilla and Biomedicine Institute of Sevilla/CSIC, Seville, Spain; 4 CIBERINFEC, Instituto de Salud Carlos III, Madrid, Spain; 5 Inserm Clinical Investigation Center 1435, Dupuytren University Hospital, Limoges, France; 6 Health Sciences North Research Institute, Sudbury, ON Canada; 7 Janssen Research and Development, Raritan, NJ USA; 8 Bacterial Vaccines Discovery and Early Development, Janssen Vaccines & Prevention B.V., Leiden, The Netherlands; 9 Julius Center for Health Sciences and Primary Care, University Medical Center Utrecht, Utrecht, The Netherlands; 10 Laboratory of Medical Microbiology, Vaccine and Infectious Disease Institute, University of Antwerp, Antwerp, Belgium; 11 Department of Respiratory Medicine, Okitama Public General Hospital, 2000, Nishi-Otsuka, Kawanishi, Yamagata Japan; 12 Department of General Internal Medicine, National Hospital Organization Nagasaki Medical Center, Nagasaki, Japan; 13 Division of Infectious Diseases, Department of Diagnostic and Public Health, University of Verona, Verona, Italy; 14 Department I of Internal Medicine, Faculty of Medicine and University Hospital of Cologne, University of Cologne, 50924 Cologne, Germany; 15 German Centre for Infection Research, Partner Site Bonn-Cologne, Cologne, Germany; 16 Department of Medicine, Division of Infectious Diseases, Duke University School of Medicine, Durham, NC USA; 17 Janssen Vaccines, Branch of Cilag GmbH International, Bern, Switzerland; 18 Department of Medical Microbiology, University Medical Center Utrecht, Utrecht, The Netherlands; 19 Present Address: European and Developing Countries Clinical Trials Partnership (EDCTP), Brussels, Belgium

**Keywords:** Invasive *E. coli* disease, Sepsis, Bacteremia, Bloodstream infection, Extraintestinal pathogenic *E. coli*, Antimicrobial resistance, Elderly

## Abstract

**Background:**

Clinical data characterizing invasive *Escherichia coli* disease (IED) are limited. We assessed the clinical presentation of IED and antimicrobial resistance (AMR) patterns of causative *E. coli* isolates in older adults.

**Methods:**

EXPECT-2 (NCT04117113) was a prospective, observational, multinational, hospital-based study conducted in patients with IED aged ≥ 60 years. IED was determined by the microbiological confirmation of *E. coli* from blood; or by the microbiological confirmation of *E. coli* from urine or an otherwise sterile body site in the presence of requisite criteria of systemic inflammatory response syndrome (SIRS), Sequential Organ Failure Assessment (SOFA), or quick SOFA (qSOFA). The primary outcomes were the clinical presentation of IED and AMR rates of *E. coli* isolates to clinically relevant antibiotics. Complications and in-hospital mortality were assessed through 28 days following IED diagnosis.

**Results:**

Of 240 enrolled patients, 80.4% had bacteremic and 19.6% had non-bacteremic IED. One-half of infections (50.4%) were community-acquired. The most common source of infection was the urinary tract (62.9%). Of 240 patients, 65.8% fulfilled ≥ 2 SIRS criteria, and 60.4% had a total SOFA score of ≥ 2. Investigator-diagnosed sepsis and septic shock were reported in 72.1% and 10.0% of patients, respectively. The most common complication was kidney dysfunction (12.9%). The overall in-hospital mortality was 4.6%. Of 299 *E. coli* isolates tested, the resistance rates were: 30.4% for trimethoprim-sulfamethoxazole, 24.1% for ciprofloxacin, 22.1% for levofloxacin, 16.4% for ceftriaxone, 5.7% for cefepime, and 4.3% for ceftazidime.

**Conclusions:**

The clinical profile of identified IED cases was characterized by high rates of sepsis. IED was associated with high rates of AMR to clinically relevant antibiotics. The identification of IED can be optimized by using a combination of clinical criteria (SIRS, SOFA, or qSOFA) and culture results.

**Supplementary Information:**

The online version contains supplementary material available at 10.1007/s15010-023-02163-z.

## Introduction

*Escherichia coli* is a ubiquitous gram-negative species that has a wide range of genetic lineages, which can sometimes allow it to cause pathogenic disease [1]. Extraintestinal pathogenic *E. coli* (ExPEC) is the most common gram-negative bacterial pathogen in humans that can move out the gastrointestinal (GI) tract and infect otherwise sterile parts of the body leading to invasive *E. coli* disease (IED) [2, 3]. IED is defined as an acute illness consistent with systemic bacterial infection, which is microbiologically confirmed either by the isolation and identification of *E. coli* from blood or other sterile body sites, or by the isolation and identification of *E. coli* from urine in patients with urosepsis [3]. *E. coli* is the most frequently identified pathogen in the etiology of bloodstream infections (BSI) and urinary tract infections (UTI) globally [4], and is the most common pathogen causing sepsis in the United States [5]. Epidemiological studies conducted over the past 2 decades reveal marked temporal increases in the number of *E. coli* BSI, including an increase of 71% in Europe [6], and the regional temporal increases in many developed countries [7–12].

These observations can be attributed to the population aging, the rising prevalence of antibiotic-resistant *E. coli* isolates, and the shifting epidemiology toward community-onset infections [10–12]. Disproportionately higher incidence rates of *E. coli* bacteremia per 100,000 person-years were reported in older adults aged 60–69 years (110 episodes) and ≥ 80 years (319 episodes) relative to the population average (48 episodes) by a systematic review covering multinational populations [13]. The treatment of older patients with IED might be complicated by the rapidly spreading drug-resistant *E. coli* strains such as sequence type 131 [14]. *E. coli* can be resistant to clinically relevant antimicrobial agents including fluoroquinolones, extended-spectrum cephalosporins, and trimethoprim-sulfamethoxazole [1, 4, 15], with *E. coli–*resistant infections being associated with a significantly higher mortality compared with that of susceptible infections [16]. Accordingly, a systematic analysis revealed that *E. coli* was among the leading pathogens contributing to the global mortality burden associated with antimicrobial resistance (AMR) in 2019 [17].

Diagnosing IED poses a challenge due to the diversity of its clinical features. A case definition of IED based on culture results and clinical criteria has been proposed to improve consistency in capturing a broad spectrum of disease states associated with IED and to help quantify the attributable disease burden [2, 3]. This improvement in consistently identifying IED is important to ensure vaccine candidates are effective against the most problematic isolates and to also address the key unmet needs for patients with IED.

In this study (EXPECT-2; NCT04117113), we used this composite case definition to identify IED among a prospectively enrolled multinational cohort of hospitalized adults aged ≥ 60 years. The primary objectives were to characterize the clinical presentation of IED and the AMR patterns of causative *E. coli* isolates in this population. In addition, we evaluated the proportion of patients with a history of a medical condition considered to carry an increased risk of IED and the AMR patterns among patients stratified by the presence of sepsis and mortality. Our findings informed the implementation of a phase 3 trial testing the efficacy of a novel vaccine candidate (ExPEC9V) for the prevention of IED in older adults with a history of UTI in the previous 2 years (NCT04899336) [18].

## Methods

### Study design and setting

This was a prospective, multicenter, hospital-based, observational study conducted at 8 sites in 7 countries (United States, Canada, France, Germany, Italy, Spain, and Japan [2 sites]). The study was initiated on October 22, 2019 (the date when the first site started data collection) and was completed on January 28, 2021 (the date of the last data collection time point for the last included patient) and included the period overlapping with the occurrence of the coronavirus disease-2019 (COVID-19) pandemic. Prospective data collection included demographics and clinical data including microbiological data. Clinical data were collected on day 1 of IED diagnosis (the day of appearance of first IED signs/symptoms) and at follow-up (discharge or day 28 after diagnosis in patients who remained hospitalized, whichever occurred first). Data on outcome of IED were collected at follow-up. This study is reported according to the Strengthening the Reporting of Observational Studies in Epidemiology (STROBE) guidelines for cohort studies (Additional file 1: STROBE Checklist).

### Population

Patients meeting the following criteria were eligible to participate: (1) aged ≥ 60 years, (2) hospitalized, (3) had a culture confirmation of *E. coli* from a normally sterile body site (including blood), and/or from urine in the presence of systemic inflammatory response syndrome (SIRS), sepsis, or septic shock consequent to the infection (see section IED Definitions); and (4) a signed participation agreement form (or an informed consent form, informed assent form, or a non-opposition form in France). Informed consent form was waived by the Ethics Committee/Institutional Review Board for eligible patients in Canada and United States. For deceased patients, participation agreement was signed by the patient’s next of kin. The study had no exclusion criteria.

Patient screening was performed by prospectively monitoring microbiological data in the laboratories of participating hospitals. Patients with an *E. coli*–positive culture were evaluated for the presence of IED (see Clinical Case Identification of IED). A list of *International Classification of Diseases* codes that could be used to identify suspected IED cases was supplied to the study sites (Additional file 2: Table S1). If a patient was hospitalized more than once during the data collection period, only the initial occurrence of IED was captured. Data collection was completed after all available data were recorded in the case report form. Only data available under normal clinical practice and stored samples were collected; no additional data were requested from individual patients. A patient was considered withdrawn from data collection following their withdrawal of consent.

### Outcomes

The following outcomes were evaluated as numbers and proportions of: (1) patients with bacteremic and non-bacteremic IED; (2) IED patients with a history of selected medical conditions potentially associated with an increased risk of IED (Additional file 3: Table S2); (3) IED patients stratified by the infection acquisition setting and by the source of infection; (4) clinical features of IED: patients fulfilling the minimum requirements of SIRS, Sequential Organ Failure Assessment (SOFA), and quick SOFA (qSOFA); investigator-diagnosed sepsis and septic shock; signs and symptoms of UTI; IED complications; (5) outcome of IED at follow-up; (6) treatment of IED; (7) AMR of causative *E. coli* isolates to antibiotics clinically relevant for the treatment of IED [15]: extended-spectrum cephalosporins (cefepime, ceftazidime, ceftriaxone), fluoroquinolones (ciprofloxacin, levofloxacin), and trimethoprim-sulfamethoxazole. These outcomes were explored in the analysis of patients stratified by bacteremic versus non-bacteremic IED and by the infection acquisition setting, where relevant.

### Data sources and outcome measurement

Data sources were the hospital records of each patient from the study site including any clinical and microbiology data. Additional data sources included laboratory results of the antibiotic susceptibility testing.

#### Clinical case identification of IED

A patient was labeled as having IED if the following criteria were met: *E. coli* in blood or in any other sterile body site (e.g. cerebrospinal fluid, pleura) or *E. coli* in urine (colony-forming units [CFU]/mL ≥ 10^3^) with no other identifiable site of infection than the urinary tract in the presence of clinical criteria, i.e. a qSOFA score of ≥ 2, or a total SOFA score of ≥ 2 points, or fever > 38 °C (100.4 °F), or hypothermia < 36 °C (96.8 °F), or ≥ 2 of the clinical criteria listed in Additional file 4: Table S3.

#### IED definitions

An IED was considered bacteremic if *E. coli* was culture-confirmed in the blood. Otherwise, the IED was considered non-bacteremic. Non-bacteremic IED was determined by the isolation and identification of *E. coli* from urine or an otherwise sterile body site in the absence of a positive blood culture and in the presence of requisite clinical criteria (SIRS, SOFA, or qSOFA). When multiple *E. coli–*positive samples were available (i.e. positive blood sample and another positive urine or sterile-site sample), the patient was classified as bacteremic IED. The IED was characterized according to the infection acquisition setting as a community-acquired, hospital-acquired, or healthcare-associated infection (Additional file 5: Table S4) and by the source of infection, which was defined by the presence of an infectious focus within 30 days before IED as determined by investigator.

#### Sepsis and septic shock

Sepsis and septic shock were diagnosed by investigator; additionally, diagnoses of sepsis were assessed programmatically in accordance with Sepsis-3 guidelines [19].

#### Complications

Complications were classified as follows: any infectious complication, kidney dysfunction, hypotension, heart dysfunction, lung dysfunction, disseminated intravascular coagulation, brain dysfunction, hypoperfusion, pneumonia, hepatic dysfunction, and other.

#### Isolate collection

All clinical samples were obtained according to routine local procedures and were considered study samples once the patient signed the informed consent form. Sample processing was conducted in accordance with the local standard procedures. Isolates were collected following inclusion of a patient and the confirmation of *E. coli* in culture and were stored and prepared for shipment to the central laboratory through microbanks (provided by the central laboratory to the local laboratories) in line with the study procedures.

#### Sample processing

Upon receipt of samples in the local laboratory, the culture test, the identification test, the susceptibility test, and the temporary isolates preservation were performed. Culture testing was performed according to the standard procedures of the local laboratory. The results were reported to the clinician via the regular path of communication at the site and used to inform clinical decision-making without delay. Identification of isolated pathogens and antimicrobial susceptibility testing (AST) were performed according to local routine laboratory procedures. A qualified member of the clinical team was responsible for entering microbiology data (including AST profile) into the electronic case report form. Urine quantitative culture result units, in terms of *E. coli*, were specified as CFU/mL.

#### Shipment of isolates and the central laboratory procedures

For confirmation of resistance after local testing, the study isolates were stored at – 80 °C and shipped to the central laboratory (University of Antwerp, Belgium) according to instructions provided by the sponsor under frozen conditions using dry ice. The shipments of study isolates were organized by the central laboratory and were performed with dry ice. Isolates remained stored at – 80 °C until further instruction was received from the central laboratory. Locally collected *E. coli* isolates were received and re-identified by the central laboratory after subculturing. If *E. coli* species identity could not be confirmed, isolates were removed from the study unless a back-up isolate with a positive *E. coli* identification result was provided by the site.

#### Antimicrobial susceptibility testing

AST was performed by the central laboratory according to the broth microdilution assay in accordance with Clinical and Laboratory Standards Institute (CLSI) guidelines, with interpretations regarding susceptibility or resistance based on CLSI-established breakpoints (30th Edition, 2020) [20]. Colistin minimal inhibitory concentration was interpreted according to the established breakpoints of European Committee on Antimicrobial Susceptibility Testing (EUCAST; version 11, 2021) [21]. Data obtained from AST were uploaded from the central laboratory into the sponsor’s database.

#### Data quality assurance

Steps taken to ensure the accuracy and reliability of data included selecting qualified study site staff and appropriate study sites, reviewing data collection procedures, remote medical monitoring, periodic centralized monitoring visits by the sponsor, and ongoing remote monitoring by the local clinical research associate. Detailed operational procedures regarding the preparation, handling, culture, and storage of clinical and microbial specimens were requested before study sites were selected.

### Statistical analyses

The study was descriptive and not powered to perform formal hypothesis testing. The study was designed to collect data from 240 patients in 8 countries for approximately 12 months (30 patients per country) and to analyze approximately 320 isolates from 240 patients. The COVID-19 pandemic limited the availability of local research resources. No patients were recruited in the UK site due to long contracting processes. Demographics and clinical characteristics were described using tabulations (numbers, proportions, and 95% confidence intervals [CIs]) for categorical variables and descriptive statistics (number of observations, mean, 95% CI [if applicable], standard deviation [SD], median, interquartile range, and range) for continuous variables. No imputations were performed to manage missing data.

## Results

### Population

**Fig. 1:**
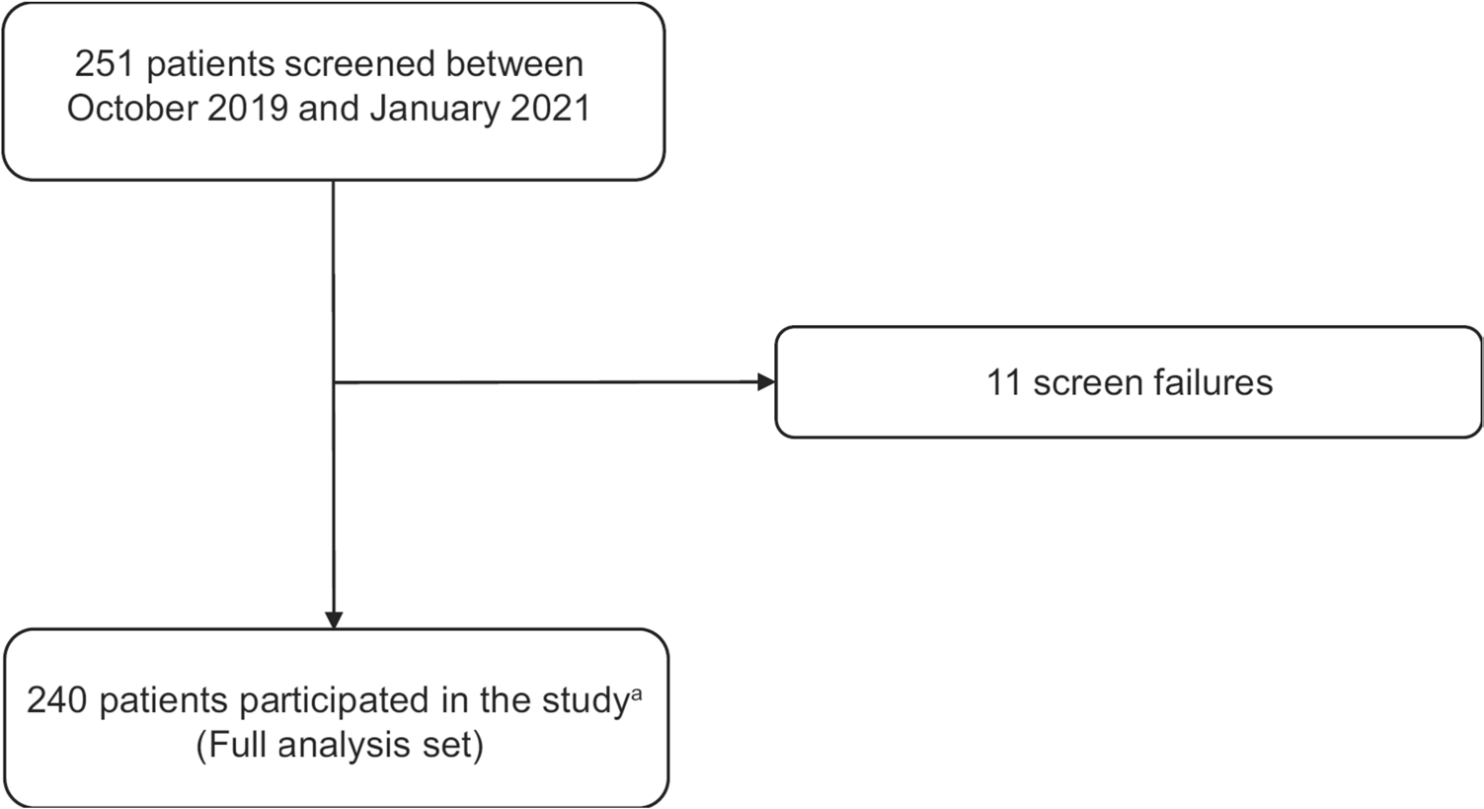
Study flow diagram. ^a^*E. coli* isolates that were sent to the central laboratory were available in 238 patients: one sample was discarded accidentally, and another was removed after an absence of *E. coli* was discovered upon whole genome sequencing

The primary set for all outcome analyses included patients meeting the eligibility criteria (full analysis set [FAS]). From 22 October 2019 through 28 January 2021, 251 patients were screened, and 240 patients participated in the study (FAS) (Fig. 1). This study period overlapped with the COVID-19 pandemic, during which enrollment was paused and restarted in 3 of 8 participating study sites (in Italy, the USA, and France), and delayed in another (in the UK). This delay eventually prevented recruitment at this site, as by October 2020 its clinical team no longer had the capacity to identify potential participants. The study was completed despite the disruption, and its primary and secondary objectives were achieved. Overall, the impact of the COVID-19 pandemic was considered limited.

Median age (range) was 75.0 years (60–97); 50.8% of patients were female (Table 1). A higher number of patients were recruited in Spain and France to compensate for the lack of recruitment in the UK. A country-level distribution of IED episodes and positive *E. coli* isolates is shown in Additional file 6: Table S5.

**Table 1:** Baseline characteristics of patients with invasive IED stratified by bacteremic and non-bacteremic IED (FAS)

	Bacteremic IED	Non-bacteremic IED	All IED
Analysis set: FAS	193	47	240
Age at the time of diagnosis (years), n	193	47	240
Median	74.0	75.0	75.0
Range (min, max)	(60, 97)	(63, 96)	(60, 97)
Age category, n (%)			
60–74	97 (50.3)	20 (42.6)	117 (48.8)
75–84	61 (31.6)	18 (38.3)	79 (32.9)
≥ 85	35 (18.1)	9 (19.1)	44 (18.3)
Sex, n (%)	193	47	240
Female	96 (49.7)	26 (55.3)	122 (50.8)
Male	97 (50.3)	21 (44.7)	118 (49.2)
Race, n (%)	138	29	167
African	4 (2.9)	3 (10.3)	7 (4.2)
Asian	26 (18.8)	4 (13.8)	30 (18.0)
Hispanic or Latino	0	1 (3.4)	1 (0.6)
Indian	1 (0.7)	0	1 (0.6)
White	107 (77.5)	21 (72.4)	128 (76.6)
Current living status, n (%)	193	47	240
At home	178 (92.2)	42 (89.4)	220 (91.7)
Long-term care facility	12 (6.2)	5 (10.6)	17 (7.1)
Other	3 (1.6)	0	3 (1.3)
Traveled outside home country (6 mo before IED), n (%)	193	47	240
Yes	3 (1.6)	1 (2.1)	4 (1.7)
No	167 (86.5)	26 (55.3)	193 (80.4)
Unknown	23 (11.9)	20 (42.6)	43 (17.9)
Medication use (3 mo before IED), n (%)	193	47	240
Antibiotics	73 (37.8)	17 (36.2)	90 (37.5)
Immunosuppressive therapy^a^	54 (28.0)	4 (8.5)	58 (24.2)
Underlying conditions (≥ 15% of patients), n (%)			
Cardiovascular disease	124 (64.2)	30 (63.8)	154 (64.2)
Urinary tract infection	67 (34.7)	23 (48.9)	90 (37.5)
Diabetes mellitus	63 (32.6)	18 (38.3)	81 (33.8)
Malignancy	69 (35.8)	12 (25.5)	81 (33.8)
Gastrointestinal disease	31 (16.1)	8 (17.0)	39 (16.3)
Urinary catheterization	33 (17.1)	4 (8.5)	37 (15.4)
Chronic kidney disease	31 (16.1)	4 (8.5)	35 (14.6)
Chronic obstructive pulmonary disease	24 (12.4)	11 (23.4)	35 (14.6)

*FAS* full analysis set, *IED* invasive *Escherichia coli* disease, *UTI* urinary tract infection

^a^Immunosuppressive therapy included steroids, anticancer chemotherapy, radiation therapy, or cytotoxic drugs

### Characterization of IED

Of 240 IED episodes, 80.4% were bacteremic and 19.6% were non-bacteremic. Baseline characteristics were balanced between bacteremic and non-bacteremic IED (Table 1). The most commonly reported medical history conditions were cardiovascular disease (64.2%]), UTI (37.5%), diabetes mellitus (33.8%), malignancy (33.8%), GI disease (16.3%) and urinary catheterization (15.4%) (Table 1 and Additional file 7: Table S6).

Half of the infections (50.4%) were community-acquired; 29.6% of infections were healthcare-associated, and 20.0% were hospital-acquired. The proportions of IED cases originating from community setting (49.2% vs. 55.3%), healthcare-associated setting (30.6% vs. 25.5%), and hospital setting (20.2% vs. 19.1%) were similar between bacteremic and non-bacteremic IED. Cardiovascular disease was more prevalent comorbidity among IED patients with community-acquired (75.9%) and healthcare-associated infections (76.8%) than with hospital-acquired infections (44.2%). A similar trend was observed for diabetes mellitus with the prevalence of 40.7% and 44.9% among community-acquired and healthcare-associated infections, respectively, versus 14.0% among hospital-acquired infections (Additional file 8: Table S7). In this latter group, the highest rate of preexisting malignancy was observed (55.8%). Within 3 months before IED onset, the use of immunosuppressors was approximately twice as frequent with hospital-acquired (54.5%) and healthcare-associated infections (68.2%) compared with community-acquired infections (27.8%), whereas the use of antibiotics was similar across infection acquisition settings and ranged between 70.5% and 86.1%.

The most common source of infection was the urinary tract (62.9%), followed by intraabdominal infection (20.4%), other (14.2%), and respiratory tract (2.5%). The urinary tract was a more common source of infection with non-bacteremic IED (93.6% [44/47]) than with bacteremic IED (55.4% [107/193]), whereas the intraabdominal infection was a more common source of infection with bacteremic IED (24.9% [48/193]) than with non-bacteremic IED (2.1% [1/47]).

### Clinical presentation of IED

Of 240 patients with IED (Table 2), 65.8% fulfilled ≥ 2 criteria of SIRS, 60.4% had a total SOFA score of ≥ 2 points, and 9.6% had a qSOFA score of ≥ 2. Furthermore, 9.2% of patients fulfilled none of the SIRS criteria. Investigator-diagnosed sepsis and septic shock were reported in 72.1% and 10.0% of patients, respectively. Over four-fifths (195/240; 81.3%) had one or more laboratory values indicating bacterial infection and/or sepsis, including changed white blood cell or platelet counts, prothrombin time, activated partial thromboplastin time, bilirubin and/or creatinine. Indeed, over half (129/240; 53.8%) of patients had leukocytosis, leukopenia or bandemia. One or more signs/symptoms of UTI were reported in 50.4% of patients. When considering patients with a urinary source of infection (151/240), the signs/symptoms of UTI were more common in patients with non-bacteremic IED (75.0% [33/44]) than in patients with bacteremic IED (59.8% [64/107]). Likewise, patients with community-acquired IED experienced one or more signs/symptoms of UTI more frequently (63.3%) than patients with hospital-acquired (34.8%) or healthcare-associated IED (40.8%) (Additional file 9: Table S8). However, in general, the signs and symptoms of IED were observed at comparable rates between bacteremic and non-bacteremic IED (Additional file 9: Table S8).

**Table 2:** Clinical presentation of patients with invasive IED by bacteremic and non-bacteremic IED (FAS)

	Bacteremic IED	Non-bacteremic IED	All IED
Analysis set: FAS	193	47	240
Any general symptom of IED^a,A^, n (%)	113 (58.5)	29 (61.7)	142 (59.2)
Any laboratory values indicating bacterial infection and/or sepsis^b,A^, n (%)	166 (86.0)	29 (42.0)	195 (81.3)
Diarrhea^A^, n (%)	11 (5.7)	3 (6.4)	14 (5.8)
SIRS evaluated, n (%)			
0 criteria	19 (9.8)	3 (6.4)	22 (9.2)
≥ 1 criterion	174 (90.2)	44 (93.6)	218 (90.8)
≥ 2 criteria	125 (64.8)	33 (70.2)	158 (65.8)
≥ 3 criteria	60 (31.1)	14 (29.8)	74 (30.8)
4 criteria	9 (4.7)	2 (4.3)	11 (4.6)
SIRS criteria			
Tachycardia^c^, n	190	47	237
Tachycardia (present), n (%)	109 (57.4)	31 (66.0)	140 (59.1)
Tachypnea^d^, n	120	44	164
Tachypnea (present), n (%)	41 (34.2)	17 (38.6)	58 (35.4)
Abnormal temperature^e^, n	187	46	233
Abnormal temperature (present), n (%)	108 (57.8)	26 (56.5)	134 (57.5)
Leukocytosis, leukopenia, or bandemia^f^, n	193	47	240
Leukocytosis, leukopenia, or bandemia (present), n (%)	110 (57.0)	19 (40.4)	129 (53.8)
Total SOFA score^g^, n (%)	193	47	240
Score ≥ 2	120 (62.2)	25 (53.2)	145 (60.4)
Score < 2	73 (37.8)	22 (46.8)	95 (39.6)
Mean (SD)	2.57 (2.106)	1.89 (1.784)	2.44 (2.061)
Q1, median, Q3	1, 2, 4	0, 2, 3	1, 2, 4
Range (min, max)	0, 9	0, 7	0, 9
qSOFA score^h^, n (%)			
Score 0	137 (71.0)	22 (46.8)	159 (66.3)
Score 1	43 (22.3)	15 (31.9)	58 (24.2)
Score 2	13 (6.7)	10 (21.3)	23 (9.6)
Score 3	0	0	0 (0)
Sepsis^A^, n (%)	138 (71.5)	35 (74.5)	173 (72.1)
Septic shock^A^, n (%)	21 (10.9)	3 (6.4)	24 (10.0)
UTI (one or more signs/symptoms)^A^, n (%)	88 (45.6)	33 (70.2)	121 (50.4)
Nausea/vomiting	29 (15.0)	7 (14.9)	36 (15.0)
Dysuria	20 (10.4)	7 (14.9)	27 (11.3)
Hematuria	16 (8.3)	10 (21.3)	26 (10.8)
Flank pain	20 (10.4)	5 (10.6)	25 (10.4)
Suprapubic pain/tenderness	13 (6.7)	10 (21.3)	23 (9.6)
Pyuria	13 (6.7)	9 (19.1)	22 (9.2)
Urgency and/or frequency to void	11 (5.7)	7 (14.9)	18 (7.5)
Straining to void	8 (4.1)	2 (4.3)	10 (4.2)
Small voids	3 (1.6)	0	3 (1.3)
Other	11 (5.7)	6 (12.8)	17 (7.1)
Complications of IED, n (%)	40	8	48
Kidney dysfunction	25 (13.0)	6 (12.8)	31 (12.9)
Hypotension	10 (5.2)	2 (4.3)	12 (5.0)
Heart dysfunction	6 (3.1)	0	6 (2.5)
Lung dysfunction	5 (2.6)	0	5 (2.1)
Disseminated intravascular coagulation	4 (2.1)	0	4 (1.7)
Brain dysfunction	2 (1.0)	0	2 (0.8)
Hypoperfusion	2 (1.0)	0	2 (0.8)
Pneumonia	2 (1.0)	0	2 (0.8)
Hepatic dysfunction	1 (0.5)	0	1 (0.4)
Other	14 (7.3)	0	14 (5.8)

*FAS* full analysis set, *IED* invasive *Escherichia coli* disease, *Paco*_*2*_ partial pressure of carbon dioxide, *SIRS* systemic inflammatory response syndrome, *SOFA* Sequential Organ Failure Assessment, *UTI* urinary tract infection, *WBC* white blood cell

^a^General symptoms of IED are malaise, fatigue, muscle pain, or chills

^b^Any laboratory values indicating an important bacterial infection and/or sepsis, including, but not limited to, white blood cell count or immature bands, platelets, prothrombin time, activated partial thromboplastin time, bilirubin, creatinine

^c^Heart rate > 90 beats per min

^d^Respiratory rate > 20 breaths per min or arterial carbon dioxide tension, Paco_2_ < 32 mm Hg

^e^Body temperature > 38 °C (100.4 °F) or hypothermia (body temperature < 36 °C [96.8 °F])

^f^Leukocytosis: WBC > 12,000 cells/mm^3^ (or 12 × 10^9^ cells/L); or leukopenia: WBC < 4,000 cells/mm^3^ (or 4 × 10^9^ cells/L); or bandemia > 10% band cells

^g^A SOFA score ≥ 2 indicates the presence of sepsis

^h^A qSOFA score ≥ 2 indicates that the patient is likely to be septic

^A^Patients may appear in ≥ 1 category

One or more complications of IED were reported in 20.0% (48/240) of patients (median duration, 4 days [IQR 2–10]). The most common complication was kidney dysfunction (12.9%; [31/240]) (Table 2). A diagnostic or interventional procedure associated with invasive disease was performed in 65.0% of patients.

### Clinical outcome and treatment of IED

Through 28 days of follow-up, 206 patients were discharged (85.8%), 23 were hospitalized (9.6%), and 11 died (4.6%), including 6 patients with bacteremic IED and 5 patients with non-bacteremic IED. One patient with non-bacteremic IED died from COVID-19. All patients received antibiotic therapy for IED at the time of study inclusion. The most commonly used antibiotics (≥ 10% of patients) were ciprofloxacin (50.8%), followed by piperacillin/tazobactam (27.9%), piperacillin (14.6%), amoxicillin-clavulanate (11.7%), meropenem (11.7%), and vancomycin (10.0%) (Additional file 10: Table S9).

### Microbiological characterization of IED

Samples from blood, urine or other sterile sites were available for 240 patients. The number of samples yielding *E. coli* was 334; 209 (62.6%) were blood, 119 (35.6%) were urine, and 6 (1.8%) were sterile-site samples. *E. coli* was the only pathogen in 82.6% of samples and was detected alongside other pathogens in 17.4% of samples. The most common co-occurring pathogens were *Enterococcus faecium* (22.4% [13/58]; all in patients with bacteremic IED), *Enterococcus faecalis* (12.1% [7/58]; 5 in patients with bacteremic IED and 2 in patients with non-bacteremic IED), and *Klebsiella pneumoniae* (10.3% [6/58]; all in patients with bacteremic IED). Of 96 urine samples meeting the criterion of ≥ 10^3^ CFU/mL, 64.6% fulfilled the criterion of ≥ 10^5^ CFU/mL.

### Antimicrobial resistance of causative *E. coli* isolates

Of 304 *E. coli* culture–positive isolates that were sent to the central laboratory, 5 isolates were deemed duplicates and discarded. Among 299 isolates from 238 patients (Fig. 1) included in AST, the resistance rates were 30.4% for trimethoprim-sulfamethoxazole, 22.1% for levofloxacin, 24.1% for ciprofloxacin, 4.3% for ceftazidime, 5.7% for cefepime, and 16.4% for ceftriaxone (Table 3). No resistance was found among 7 evaluated last-resort antibiotics (ceftazidime/avibactam, ceftolozane/tazobactam, doripenem, ertapenem, imipenem, and meropenem). When analyzing isolates collected in 11 patients who died (12 isolates), increased resistance rates were observed for trimethoprim-sulfamethoxazole (41.7%), levofloxacin (33.3%) and ciprofloxacin (33.3%) (Additional file 11: Table S10). However, the resistance rates were comparable between the 179 isolates collected from 143 patients with septic IED and 120 isolates from 95 patients with non-septic IED (Additional file 12: Table S11).

**Table 3:** Number and percentage of resistant *E. coli* isolates collected from patients with invasive IED (FAS)

	All IED
Analysis set: FAS	238
Number of *E. coli* isolates with AST performed	299
Number of *E. coli* isolates resistant to a given antibiotic^abc^, (%)	
Amikacin	2 (0.7)
Ampicillin	168 (56.2)
Ampicillin-sulbactam	56 (18.7)
Aztreonam	27 (9.0%)
Cefazolin	56 (18.7)
Cefepime	17 (5.7)
Ceftazidime	13 (4.3)
Ceftriaxone	49 (16.4)
Ciprofloxacin	72 (24.1)
Colistin	3 (1.0)
Gentamicin	33 (11.0)
Levofloxacin	66 (22.1)
Minocycline	18 (6.0)
Piperacillin-tazobactam	5 (1.7)
Tetracycline	91 (30.4)
Tigecycline	1 (0.3)
Tobramycin	30 (10.0)
Trimethoprim-sulfamethoxazole	91 (30.4)
Number of *E. coli* isolates resistant to antibiotic by class, (%)	
≥ 1 antibiotic in ≥ 1 drug class	186 (62.2)
≥ 1 antibiotic in ≥ 2 drug classes	136 (45.5)
≥ 1 antibiotic in ≥ 3 drug classes	104 (34.8)

Antibiotic drug classes evaluated: aminoglycoside, carbapenem, cephalosporin, fluoroquinolone, folate pathway inhibitor(s), fosfomycin, nitrofurantoin, penicillin, penicillin/β-lactamase inhibitor, polymyxin/lipopeptide, and tetracycline. Interpretations regarding susceptibility or resistance were reported according to CLSI and EUCAST established breakpoints (the latter was used only for colistin MIC interpretation) [20, 21]

*AST* antimicrobial susceptibility test, *CLSI* Clinical and Laboratory Standards Institute, *EUCAST* European Committee on Antimicrobial Susceptibility Testing, *MIC* minimal inhibitory concentration

^a^Denominator is total number of *E. coli* isolates with AST performed

^b^A patient may have more than one isolate test result

^c^None of the *E. coli* isolates were resistant to the following antibiotics: ceftazidime-avibactam, ceftolozane-tazobactam, doripenem, ertapenem, imipenem, meropenem and nitrofurantoin

## Discussion

This study described the clinical features of IED and AMR patterns of causative *E. coli* isolates among prospectively enrolled, hospitalized adults aged ≥ 60 years. We defined IED as a bacterial infection with acute systemic consequences by using a composite clinical and microbiological criteria, including the clinical evaluation against the requisite scores of SIRS, SOFA, and qSOFA and a microbiological confirmation of ≥ 1 *E. coli* isolate cultured from blood, urine, or an otherwise sterile body site [2, 3]. This definition allowed us to differentiate between bacteremic and non-bacteremic IED and to perform a fine-grained stratification of IED according to the infection acquisition setting and the source of infection.

IED was bacteremic in the majority of patients (80.4%). The most common culture-positive site was blood (62.6%). In line with these observations, a retrospective multicenter cohort study of 902 predominantly elderly patients with IED reported that 77.9% of *E. coli* isolates originated from blood [2]. In this study, approximately one-half of infections were community-acquired (50.4%), whereas hospital-acquired infections were markedly less frequent (20.0%). This finding is corroborated by international and nationwide epidemiologic data showing the predominance of community-acquired *E. coli* BSI [8, 22–24], and substantially lower reported rates of hospital-onset invasive *E. coli* infections [22, 25]. In addition, the urinary tract was the most common primary source of infection (62.9%), consistent with the data reported in a systematic review documenting the urinary tract as the leading source of *E. coli* bacteremia [13], and with the data revealing higher prevalence of BSIs originating from the urinary tract in older versus younger populations [26].

Sepsis and septic shock can be considered to be the most severe forms of IED. In this study, the rates of investigator-diagnosed sepsis and septic shock were 72.1% and 10.0%, respectively. These observations contrast with the relatively favorable patient outcomes observed through 28 days of follow-up, with 9.6% of patients remaining hospitalized and the in-hospital mortality rate of 4.6%. These outcomes are at odds with the high sepsis-related mortality reported in the literature [27–29]. For example, the average 30-day sepsis mortality rate was 24.4% and increased to 34.7% with the presence of shock according to the systematic review and meta-analysis [27]. Furthermore, a retrospective cohort data from 902 patients with IED, including 65.3% of those diagnosed with sepsis, reported the case fatality rate of 20.0% that increased among older patients up to 22.2% [2]. In addition, other forms of IED such as *E. coli* bacteremia are associated with mortality ranging between 8.0% and 20.0% [2, 7, 13, 22, 30]. The relatively low observed mortality in this study might be due to the preponderance of infections with a urinary focus of attention, which tend to be associated with lower mortality rates than infections in other sites [31]. Alternatively, it could be attributed to the selection bias toward enrolling less severe IED cases in countries where informed consent was an eligibility requirement. As the waiver for informed consent was only obtained for patients enrolled in the United States and Canada, other sites might have enrolled a higher proportion of patients who were physically and mentally capable for consent and therefore at a lower risk of death due to IED.

The highest rate of AMR was found for trimethoprim-sulfamethoxazole (30.4%), followed by ciprofloxacin (24.1%), levofloxacin (22.1%), and ceftriaxone (16.4%). These rates are comparable with those reported in multinational and nationwide studies of invasive *E. coli* infections [2, 22, 32]. In addition, increased resistance rates to trimethoprim-sulfamethoxazole and fluoroquinolones were observed for isolates collected in 11 patients who died, equaling to 41.7% and 33.3%, respectively. When analyzing AMR in patients stratified by the presence of sepsis, however, comparable resistance rates with those found in the overall study population were observed. Epidemiological data on AMR of *E. coli* isolates in culture-proven sepsis shows wide variation across studies, which could be attributed to the differences in geographical coverage and bacterial etiologies. For instance, among U.S. patients hospitalized with community-acquired sepsis, the net prevalence for ≥ 1 g-negative resistant organism (including ceftriaxone-resistant pathogens, extended-spectrum β-lactamase-producing pathogens and carbapenem-resistant *Enterobacteriaceae*), was 13.2% [5]. By contrast, nationwide studies report markedly higher resistance for gram-negative pathogens (including *E. coli*) in patients with sepsis with resistance rates to broad-spectrum cephalosporins and fluoroquinolones upward of 60–90% [33–35]. In addition, quantifying the causal contribution of AMR to 30-day mortality using observational data is notoriously difficult. In a parallel-matched cohort study of 1954 patients with gram-negative infections admitted in Dutch hospitals, including 61.0% of infections caused by *E. coli*, AMR was not associated with 30-day mortality [36].

Resistance to carbapenems was not found among the 240 *E. coli* isolates. Although resistance among Enterobacteriaceae is increasing overall, this is largely found among *Klebsiella pneumoniae* isolates, whose rates of AMR exceed 25% in some European countries. In contrast, *E. coli* resistance rates against carbapenems were generally still under 1% in Europe throughout in 2022 [37].

In this study, we used a composite case definition for identifying IED [2, 3, 38] based on the culture confirmation from blood; or from urine or an otherwise sterile body site in the presence of requisite clinical criteria of SIRS, SOFA, and qSOFA. Although IED diagnosis can be justified solely on the basis of SIRS criteria combined with an *E. coli* culture–positive sterile-site sample, the addition of clinical criteria of SOFA can optimize capture of IED, especially among patients who have a positive urine sample without a positive sterile-site sample. Our data show that more than one-third of patients (34.2%) failed to meet at least two criteria of SIRS, supporting the utility of combining SIRS and SOFA assessments when evaluating patients for the presence of IED. In line with this, a recent study estimated that 86% of IED cases would have been retrospectively identified using both SOFA and SIRS criteria as opposed to 62% of cases that would have been identified based on SOFA scores alone [2]. Elderly patients might especially pose challenges with regards to the exclusive use of SIRS criteria to capture IED due to a blunted or absent fever response that occurs in approximately 20–30% of cases [39], and the lack of routine respiratory rate assessments, which are frequently neglected in hospitals [40].

Several limitations should be considered when interpreting the findings of this study. First, a selection bias in countries where informed consent was required cannot be ruled out, which might partially explain the lower mortality rate compared with those observed in other observational studies. Second, the observed high rate of bacteremic episodes might be attributed to the nonconsecutive enrollment of all the samples that had been drawn. As the sites often reported only positive samples, the enrollment might have been biased toward the inclusion of positive blood cultures leading to the under-representation of confirmed urine cultures that were required to establish the presence of urosepsis. Third, a bias might have occurred with regards to the indication of the diagnostic tests performed in the presence of suspected invasive bacterial infection. Surgeons frequently do not submit intra-abdominal samples for microbiological testing for non-severe community-acquired invasive abdominal site infections (e.g. acute cholecystitis, acute appendicitis), whereas blood culture testing might be infrequently performed for UTI and pyelonephritis. These latter two limitations might have resulted in the lower reported percentage of non-bacteremic cases. Fourth, treatment information on empirical versus targeted prescriptions were not captured in the case report form, and the lack of information on the proportion of initial treatments that were active against the causative bacteria precludes drawing links between empirical therapy and clinical outcomes. Fifth, a discrepancy in the rates of investigator-diagnosed sepsis (72.1%) and the presence of sepsis as evidenced by ≥ 2 points in the SOFA score (60.4%) might suggest a less stringent application of Sepsis-3 guidelines by the participating hospital sites. Sixth, although selection criteria were used to ensure selection of adequate study sites, the inability to randomly select study sites could have resulted in systematic and undetectable measurement errors. Finally, the lack of data on prior hospitalizations in sites other than participating hospitals might have led to misclassifying healthcare-associated infections as community-acquired.

In conclusion, we used a prospective, observational design to characterize the clinical profile of IED, using a case definition of IED based on culture results and clinical criteria, and AMR patterns of causative *E. coli* isolates in a multinational cohort of 240 hospitalized adults aged ≥ 60 years. The clinical profile of identified IED cases was characterized by high rates of investigator-diagnosed sepsis. In light of the above-mentioned limitations of the study design likely resulting in a higher representation of bacteremic IED and less severe forms of IED, the low prevalence of non-bacteremic cases (19.6%) and the relatively low rate of mortality in the overall IED population (4.6%) should be interpreted with caution. Among antibiotics deemed clinically relevant for the treatment of IED, the highest resistance rates were found for trimethoprim-sulfamethoxazole (30.4%), ciprofloxacin (24.1%), levofloxacin (22.1%), and ceftriaxone (16.4%). The resistance to trimethoprim-sulfamethoxazole and fluoroquinolones was increased among isolates collected in patients who died. Identification of IED can be optimized by using the combination of clinical criteria (SIRS and SOFA, or qSOFA) and culture results. A combined use of SIRS and SOFA criteria could improve detection of IED, especially in the context of double-blind, placebo-controlled, randomized, phase 3 vaccine efficacy trials that aim to detect an IED endpoint in an enrolled population of elderly participants.

### Supplementary Information

Below is the link to the electronic supplementary material.

Supplementary file1 (DOCX 24 KB)

Supplementary file2 (DOCX 26 KB)

Supplementary file3 (DOCX 18 KB)

Supplementary file4 (DOCX 18 KB)

Supplementary file5 (DOCX 18 KB)

Supplementary file6 (DOCX 17 KB)

Supplementary file7 (DOCX 21 KB)

Supplementary file8 (DOCX 20 KB)

Supplementary file9 (DOCX 20 KB)

Supplementary file10 (DOCX 18 KB)

Supplementary file11 (DOCX 20 KB)

Supplementary file12 (DOCX 20 KB)

## Data Availability

Although these data are not currently publicly available for sharing, requests for sharing can be sent to the Corresponding Author and will be evaluated on an individual basis.

## Abbreviations

AMR: Antimicrobial resistance
AST: Antimicrobial susceptibility testing
BSI: Bloodstream infection
CFU: Colony-forming units
CLSI: Clinical and Laboratory Standards Institute
EUCAST: European Committee on Antimicrobial Susceptibility Testing
FAS: Full analysis set
IED: Invasive *Escherichia coli* disease
qSOFA: Quick Sequential Organ Failure Assessment
SIRS: Systemic inflammatory response syndrome
SOFA: Sequential Organ Failure Assessment
UTI: Urinary tract infection
