# Supplementary material for: Clinical presentation and antimicrobial resistance of invasive Escherichia coli disease in hospitalized older adults: a prospective multinational observational study
Source: Infection. 2024 Jan 25;52(3):1073–85. doi: 10.1007/s15010-023-02163-z (PMC11142950; doi:10.1007/s15010-023-02163-z)
Supplement: Supplementary file 2 — Supplementary file2 (DOCX 26 KB) [file 15010_2023_2163_MOESM2_ESM.docx]

**Table S1** ICD codes relevant for identifying IED

| **ICD code** | **Meaning** | **To be used in conjunction with lab codes^ab^** |
| --- | --- | --- |
| B96 | Other bacterial agents as the cause of diseases classified to other chapters | 1 to 3 |
| B96.2 | *E. coli* as the cause of diseases classified elsewhere | N/A |
| B96.20 | Unspecified *E. coli* as the cause of diseases classified elsewhere | N/A |
| B96.29 | Other *E. coli* as the cause of diseases classified elsewhere | N/A |
| A41 | Other sepsis | 1 to 3 |
| A41.5 | Sepsis due to other Gram-negative organisms | 1 to 3 |
| A41.50 | Gram-negative sepsis, unspecified | 1 to 3 |
| A41.51 | Sepsis due to *E. coli* | N/A |
| A41.8 | Other specified sepsis | 1 to 3 |
| A41.89 | Other specified sepsis | 1 to 3 |
| A41.9 | Sepsis, unspecified organism | 1 to 3 |
| A48 | Other bacterial diseases, not elsewhere classified | 1 to 3 |
| A48.8 | Other specified bacterial diseases | 1 to 3 |
| A49 | Bacterial infection of unspecified site | 1 to 3 |
| A49.8 | Other bacterial infection of unspecified site | 1 to 3 |
| A49.9 | Bacterial infection, unspecified | 1 to 3 |
| D73.3 | Abscess of spleen | 1 to 3 |
| G00 | Bacterial meningitis, not elsewhere classified | 1 to 3 |
| G00.8 | Other bacterial meningitis | 1 to 3 |
| G00.9 | Bacterial meningitis, unspecified | 1 to 3 |
| G01 | Meningitis in bacterial diseases classified elsewhere | 1 to 3 |
| G02 | Meningitis in other infectious and parasitic diseases classified elsewhere | 1 to 3 |
| G03 | Meningitis due to other and unspecified causes | 1 to 3 |
| G03.9 | Meningitis, unspecified | 1 to 3 |
| G06^⁑^ | Intracranial and intraspinal abscess and granuloma | 1 to 3 |
| I30.1 | Infective pericarditis | 1 to 3 |
| I33^⁑^ | Acute and subacute infective endocarditis | 1 to 3 |
| I38 | Endocarditis, valve unspecified | 1 to 3 |
| I39 | Endocarditis and heart valve disorders in diseases classified elsewhere | 1 to 3 |
| I77.6 | Arteritis, unspecified | 1 to 3 |
| J15 | Bacterial pneumonia, not elsewhere classified | 1 to 3 |
| J15.5 | Pneumonia due to *E. coli* | N/A |
| J15.9 | Unspecified bacterial pneumonia | 1 to 3 |
| J16.8 | Pneumonia due to other specified infectious organisms | 1 to 3 |
| J17 | Pneumonia in diseases classified elsewhere | 1 to 3 |
| J18 | Pneumonia, unspecified organism | 1 to 3 |
| J18.0 | Bronchopneumonia, unspecified organism | 1 to 3 |
| J18.1 | Lobar pneumonia, unspecified organism | 1 to 3 |
| J18.2 | Hypostatic pneumonia, unspecified organism | 1 to 3 |
| J18.8 | Other pneumonia, unspecified organism | 1 to 3 |
| J18.9 | Pneumonia, unspecified organism | 1 to 3 |
| J85^⁑^ | Abscess of lung and mediastinum | 1 to 3 |
| J86^⁑^ | Pyothorax | 1 to 3 |
| J98.51 | Mediastinitis | 1 to 3 |
| K61^⁑^ | Abscess of anal and rectal regions | 1 to 3 |
| K63.0 | Abscess of intestine | 1 to 3 |
| K65 | Peritonitis | 1 to 3 |
| K65.0 | Generalized (acute) peritonitis | 1 to 3 |
| K65.1 | Peritoneal abscess | 1 to 3 |
| K65.2 | Spontaneous bacterial peritonitis | 1 to 3 |
| K65.8 | Other peritonitis | 1 to 3 |
| K65.9 | Peritonitis, unspecified | 1 to 3 |
| K67.8 | Disorders of peritoneum in infectious diseases classified elsewhere | 1 to 3 |
| K68.1^⁑^ | Retroperitoneal abscess | 1 to 3 |
| K75.0 | Abscess of liver | 1 to 3 |
| K83.0 | Cholangitis | 1 to 3 |
| K85.8.^⁑^ | Other acute pancreatitis | 1 to 3 |
| K85.9 | Acute pancreatitis, unspecified | 1 to 3 |
| M00.8^⁑^ | Arthritis and polyarthritis due to other bacteria | 1 to 3 |
| M00.9 | Pyogenic arthritis, unspecified | 1 to 3 |
| M01^⁑^ | Direct infections of joint in infectious & parasitic diseases classified elsewhere | 1 to 3 |
| M46.3 | Infection of intervertebral disc (pyogenic) | 1 to 3 |
| M60.0 | Infective myositis | 1 to 3 |
| M71.0^⁑^ | Abscess of bursa | 1 to 3 |
| M71.1^⁑^ | Other infective bursitis | 1 to 3 |
| M72.6 | Necrotising fasciitis | 1 to 3 |
| M86^⁑^ | Osteomyelitis | 1 to 3 |
| N10 | Acute pyelonephritis | 1 to 3 |
| N11^⁑^ | Chronic tubulo-interstitial nephritis | 1 to 3 |
| N15.1 | Renal and perinephric abscess | 1 to 3 |
| N30 | Cystitis | 1 to 3^c^ |
| N30.0^⁑^ | Acute cystitis | 1 to 3^c^ |
| N30.1^⁑^ | Interstitial cystitis (chronic) | 1 to 3^c^ |
| N30.2^⁑^ | Other chronic cystitis | 1 to 3^c^ |
| N30.3^⁑^ | Trigonitis | 1 to 3^c^ |
| N30.5^⁑^ | Other cystitis | 1 to 3^c^ |
| N30.9^⁑^ | Cystitis, unspecified | 1 to 3^c^ |
| N34 ^⁑^ | Urethritis and urethral syndrome | 1 to 3^c^ |
| N39 | Urinary tract infection, site not specified | 1 to 3^c^ |
| N41.0 | Acute prostatitis | 1 to 3^c^ |
| N41.1 | Chronic prostatitis | 1 to 3^c^ |
| N41.2 | Abscess of prostate | 1 to 3^c^ |
| N41.3 | Prostatocystitis | 1 to 3^c^ |
| N43.1 | Infected hydrocele | 1 to 3 |
| N45^⁑^ | Orchitis and epididymitis | 1 to 3 |
| N70^⁑^ | Salpingitis and oophoritis | 1 to 3 |
| N76.4 | Abscess of vulva | 1 to 3 |
| O75.3 | Sepsis during labor | 1 to 3 |
| O03.37 | Sepsis following incomplete spontaneous abortion | 1 to 3 |
| O03.87 | Sepsis following complete or unspecified spontaneous abortion | 1 to 3 |
| O04.87 | Sepsis following (induced) termination of pregnancy | 1 to 3 |
| O07.37 | Sepsis following failed attempted termination of pregnancy | 1 to 3 |
| O08.82 | Sepsis following ectopic and molar pregnancy | 1 to 3 |
| O85 | Puerperal sepsis | 1 to 3 |
| O86^⁑^ | Other puerperal infections | 1 to 3 |
| O91.1^⁑^ | Abscess of breast associated with pregnancy, the puerperium and lactation | 1 to 3 |
| R09.1 | Pleurisy | 1 to 3 |
| R50 | Fever of other and unknown origin | 1 to 3 |
| R50.9 | Fever, unspecified | 1 to 3 |
| R65 | Symptoms and signs specifically associated with systemic inflammation and infection | 1 to 3 |
| R65.10 | SIRS of non-infectious origin without acute organ dysfunction | 1 to 3 |
| R65.11 | SIRS of non-infectious origin with acute organ dysfunction | 1 to 3 |
| R65.2 | Severe sepsis | 1 to 3 |
| R65.20 | Severe sepsis without septic shock | 1 to 3 |
| R65.2 | Severe sepsis with septic shock | 1 to 3 |
| R78.81 | Bacteremia | 1 to 3 |
| T80.211 | Bloodstream infection due to central venous catheter | 1 to 3 |
| T80.219 | Unspecified infection due to central venous catheter | 1 to 3 |
| T80.22 | Acute infection following infusion, transfusion and therapeutic injection | 1 to 3 |
| T81.3 | Disruption of wound, not elsewhere classified | 1 to 3 |
| T81.4 | Infection following a procedure | 1 to 3 |
| T81.89 | Other complications of procedures, not elsewhere classified | 1 to 3 |
| T81.9 | Unspecified complication of procedure | 1 to 3 |
| D78.89 | Other postprocedural complications of the spleen | 1 to 3 |
| G97.82 | Other postprocedural complications and disorders of nervous system | 1 to 3 |
| I97.89 | Other postprocedural complications and disorders of the circulatory system, not elsewhere classified | 1 to 3 |
| J95.89 | Other postprocedural complications and disorders of respiratory system, not elsewhere classified | 1 to 3 |
| K91.89 | Other postprocedural complications and disorders of digestive system | 1 to 3 |
| L76.82 | Other postprocedural complications of skin and subcutaneous tissue | 1 to 3 |
| N99.89 | Other postprocedural complications and disorders of genitourinary system | 1 to 3 |

^a^As an alternative to the ICD codes, sites may combine the clinical codes with information from the laboratory showing
a positive *E. coli* culture.

^b^Codes 1 to 3 denote:

1 = Laboratory evidence of a positive *E. coli* blood culture.
2 = Laboratory evidence of a positive *E. coli* culture from urine.

3 = Laboratory evidence of a positive *E. coli* culture from a normally sterile site.

^c^Cultured from urine.

^⁑^Plus all other lower level codes.

*ICD* International Classification of Diseases*; IED* invasive *Escherichia coli* disease; *SIRS* systemic inflammatory response syndrome.
