# Supplementary material for: Clinical presentation and antimicrobial resistance of invasive Escherichia coli disease in hospitalized older adults: a prospective multinational observational study
Source: Infection. 2024 Jan 25;52(3):1073–85. doi: 10.1007/s15010-023-02163-z (PMC11142950; doi:10.1007/s15010-023-02163-z)
Supplement: Supplementary file 3 — Supplementary file3 (DOCX 18 KB) [file 15010_2023_2163_MOESM3_ESM.docx]

**Table S2** Medical history terms considered to carry the risk for developing IED based on review of literature and data mining^a^

| Urinary tract infection |
| --- |
| Diabetes mellitus |
| Malignancy |
| Gastrointestinal disease |
| Chronic kidney disease |
| Chronic obstructive pulmonary disease |
| General weakness/poor condition |
| Urological intervention including urinary catheterization |
| Cardiovascular disease |
| Cerebrovascular accident (stroke) |
| Cholelithiasis |
| Dementia |
| Previous urosepsis |
| Chronic liver disease |
| Urinary and/or fecal incontinency |
| Obstructive uropathy |
| Cholangitis |
| Urolithiasis |
| Organ transplantation |
| Immobility |
| Cachexia |
| Neurogenic bladder |
| Anorectal conditions |
| Other |

^a^A real-world database study was conducted using electronic medical records from Kaiser Permanente Northwest in the United States (2002 to 2016), and the Clinical Practice Research Database linked to Hospital Episodes Statistics in the United Kingdom (2012 to 2017). The study identified medical conditions associated with an increased risk of IED.

*IED* invasive *Escherichia coli* disease
