# Supplementary material for: Clinical presentation and antimicrobial resistance of invasive Escherichia coli disease in hospitalized older adults: a prospective multinational observational study
Source: Infection. 2024 Jan 25;52(3):1073–85. doi: 10.1007/s15010-023-02163-z (PMC11142950; doi:10.1007/s15010-023-02163-z)
Supplement: Supplementary file 4 — Supplementary file4 (DOCX 18 KB) [file 15010_2023_2163_MOESM4_ESM.docx]

**Table S3** A list of clinical criteria used to support the case identification of IED

|  |
| --- |
| **Clinical criteria of invasive bacterial infection** |
| - Tachycardia: >90 beats per minute - Tachypnea: ≥20 breaths per minute or arterial carbon dioxide tension <32 mmHg |
| - Altered mentation (Glasgow Coma Scale score) <15 |
| - Systolic blood pressure: ≤100 mm Hg |
| - Any laboratory values indicating an important bacterial infection and/or sepsis, including, but not limited to, white blood cell count or immature bands (e.g. platelets, prothrombin time, activated partial thromboplastin time, bilirubin, creatinine) |
| - Signs and/or symptoms of urinary tract infection (e.g. dysuria, flank pain, suprapubic pain, urgency, frequency, hematuria, pyuria) |

IED, invasive *E. coli* disease
