# Supplementary material for: Clinical presentation and antimicrobial resistance of invasive Escherichia coli disease in hospitalized older adults: a prospective multinational observational study
Source: Infection. 2024 Jan 25;52(3):1073–85. doi: 10.1007/s15010-023-02163-z (PMC11142950; doi:10.1007/s15010-023-02163-z)
Supplement: Supplementary file 5 — Supplementary file5 (DOCX 18 KB) [file 15010_2023_2163_MOESM5_ESM.docx]

**Table S4** Characterization of IED by the infection acquisition setting

| **IED** | **Definition** |
| --- | --- |
| **Community-acquired infection** | An infection that occurs without exposure in the past 3 months to the risk from care in a hospital, clinic, doctor’s office, or home-care treatment by a healthcare professional |
| **Hospital-acquired infection** | Any infection that occurs during or after hospitalization that was not present or incubating at the time of the patient’s admission (>48 hours following hospital admission) |
| **Healthcare-associated infection** | An infection present at hospital admission or within 48 hours of admission in patients fulfilling any of the following criteria:   - Received intravenous therapy, wound care, or specialized nursing care at home in the previous 90 days; - Attended a hospital or hemodialysis clinic or received intravenous chemotherapy in the previous 30 days; - Were hospitalized in an acute care hospital for >1 day in the previous 90 days; - Resided in a nursing home or long-term care facility; - Immunosuppression, active or metastatic cancer, previous radiation therapy, transfer from another care facility, elderly or physically disabled persons who need healthcare, previous submission to invasive procedures, surgery performed in the last 180 days, family member with a multidrug-resistant microorganism, and recent treatment with antibiotics |

*IED* invasive *Escherichia coli* disease
