# Supplementary material for: Clinical presentation and antimicrobial resistance of invasive Escherichia coli disease in hospitalized older adults: a prospective multinational observational study
Source: Infection. 2024 Jan 25;52(3):1073–85. doi: 10.1007/s15010-023-02163-z (PMC11142950; doi:10.1007/s15010-023-02163-z)
Supplement: Supplementary file 6 — Supplementary file6 (DOCX 17 KB) [file 15010_2023_2163_MOESM6_ESM.docx]

**Table S5** Country-level distribution of IED episodes and positive *E. coli* isolates

| **Country** | **Number (%) of patients** | **Number (%) of isolates^a^** |
| --- | --- | --- |
| Canada | 30 (12.5) | 33 (11.0) |
| Germany | 30 (12.5) | 30 (10.0) |
| Spain | 45 (18.8) | 51 (17.1) |
| France | 43 (17.9) | 69 (23.1) |
| Italy | 32 (13.3) | 36 (12.1) |
| Japan | 30 (12.5) | 47 (15.7) |
| United States | 30 (12.5) | 33 (11.0) |
| All countries | 240 | 299 |

^a^Patients may have more than 1 isolate.

*IED* invasive *Escherichia coli* disease
