# Supplementary material for: Clinical presentation and antimicrobial resistance of invasive Escherichia coli disease in hospitalized older adults: a prospective multinational observational study
Source: Infection. 2024 Jan 25;52(3):1073–85. doi: 10.1007/s15010-023-02163-z (PMC11142950; doi:10.1007/s15010-023-02163-z)
Supplement: Supplementary file 7 — Supplementary file7 (DOCX 21 KB) [file 15010_2023_2163_MOESM7_ESM.docx]

**Table S6** Number and percentage of patients with IED risk-related medical history (FAS)

|  | **Bacteremic IED** | **Non-bacteremic IED** | **All IED** |
| --- | --- | --- | --- |
| Analysis set: FAS | 193 | 47 | 240 |
| Patients having history of any of the following diseases, n (%) | 189 (97.9) | 44 (93.6) | 233 (97.1) |
| Cardiovascular disease | 124 (64.2) | 30 (63.8) | 154 (64.2) |
| Urinary tract infection | 67 (34.7) | 23 (48.9) | 90 (37.5) |
| Diabetes mellitus | 63 (32.6) | 18 (38.3) | 81 (33.8) |
| Malignancy | 69 (35.8) | 12 (25.5) | 81 (33.8) |
| Gastrointestinal disease | 31 (16.1) | 8 (17.0) | 39 (16.3) |
| Urinary catheterization | 33 (17.1) | 4 (8.5) | 37 (15.4) |
| Chronic kidney disease | 31 (16.1) | 4 (8.5) | 35 (14.6) |
| COPD | 24 (12.4) | 11 (23.4) | 35 (14.6) |
| General weakness/poor condition | 22 (11.4) | 9 (19.1) | 31 (12.9) |
| Urological intervention | 23 (11.9) | 5 (10.6) | 28 (11.7) |
| Cerebrovascular accident (stroke) | 18 (9.3) | 6 (12.8) | 24 (10.0) |
| Cholelithiasis | 21 (10.9) | 0 | 21 (8.8) |
| Dementia | 14 (7.3) | 3 (6.4) | 17 (7.1) |
| Previous urosepsis | 13 (6.7) | 3 (6.4) | 16 (6.7) |
| Chronic liver disease | 13 (6.7) | 2 (4.3) | 15 (6.3) |
| Urinary incontinency | 9 (4.7) | 4 (8.5) | 13 (5.4) |
| Obstructive uropathy | 10 (5.2) | 2 (4.3) | 12 (5.0) |
| Cholangitis | 10 (5.2) | 1 (2.1) | 11 (4.6) |
| Urolithiasis | 9 (4.7) | 2 (4.3) | 11 (4.6) |
| Organ transplantation | 6 (3.1) | 0 | 6 (2.5) |
| Immobility | 2 (1.0) | 2 (4.3) | 4 (1.7) |
| Cachexia | 2 (1.0) | 1 (2.1) | 3 (1.3) |
| Fecal incontinency | 3 (1.6) | 0 | 3 (1.3) |
| Neurogenic bladder | 2 (1.0) | 1 (2.1) | 3 (1.3) |
| Anorectal conditions | 1 (0.5) | 0 | 1 (0.4) |
| Other conditions with increased risk for IED | 20 (10.4) | 2 (4.3) | 22 (9.2) |
| History of UTI (previous 10 years) |  |  |  |
| n | 184 | 47 | 231 |
| No UTI | 123 (66.8) | 25 (53.2) | 148 (64.1) |
| <2 years prior to enrollment | 40 (21.7) | 15 (31.9) | 55 (23.8) |
| ≥2 years prior to enrollment | 21 (11.4) | 7 (14.9) | 28 (12.1) |
| History of IED (previous 10 years) |  |  |  |
| n | 178 | 44 | 222 |
| No IED | 150 (84.3) | 38 (86.4) | 188 (84.7) |
| With IED | 28 (15.7) | 6 (13.6) | 34 (15.3) |

*COPD* chronic obstructive pulmonary disease, *FAS* full analysis set, *IED* invasive *Escherichia coli* disease, *UTI* urinary tract infection.
