# Supplementary material for: Clinical presentation and antimicrobial resistance of invasive Escherichia coli disease in hospitalized older adults: a prospective multinational observational study
Source: Infection. 2024 Jan 25;52(3):1073–85. doi: 10.1007/s15010-023-02163-z (PMC11142950; doi:10.1007/s15010-023-02163-z)
Supplement: Supplementary file 8 — Supplementary file8 (DOCX 20 KB) [file 15010_2023_2163_MOESM8_ESM.docx]

**Table S7** Patient characteristics stratified by the IED acquisition setting (FAS)

|  | **Community-acquired** | **Hospital-acquired** | **Healthcare-associated** | **All IED** |
| --- | --- | --- | --- | --- |
| Analysis set: Full Analysis | 121 (50.4) | 48 (20.0) | 71 (29.6) | 240 |
| Age at time of diagnosis (years) |  |  |  |  |
| Mean (SD) | 75.9 (8.53) | 71.9 (8.09) | 76.7 (8.50) | 75.4 (8.58) |
| Median | 75.0 | 71.0 | 76.0 | 75.0 |
| Range (min, max) | (61, 96) | (60, 90) | (60, 97) | (60, 97) |
| Age category, n (%) |  |  |  |  |
| 60 to 74 years | 56 (46.3) | 31 (64.6) | 30 (42.3) | 117 (48.8) |
| 75 to 84 years | 40 (33.1) | 12 (25.0) | 27 (38.0) | 79 (32.9) |
| ≥85 years | 25 (20.7) | 5 (10.4) | 14 (19.7) | 44 (18.3) |
| Sex | 121 | 48 | 71 | 240 |
| Female | 70 (57.9) | 16 (33.3) | 36 (50.7) | 122 (50.8) |
| Male | 51 (42.1) | 32 (66.7) | 35 (49.3) | 118 (49.2) |
| Relevant comorbidities | 108 | 43 | 69 | 220 |
| COPD | 16 (14.8) | 5 (11.6) | 14 (20.3) | 35 (15.9) |
| Cardiovascular disease | 82 (75.9) | 19 (44.2) | 53 (76.8) | 154 (70.0) |
| Cerebrovascular accident (stroke) | 13 (12.0) | 4 (9.3) | 7 (10.1) | 24 (10.9) |
| Cholelithiasis | 11 (10.2) | 4 (9.3) | 6 (8.7) | 21 (9.5) |
| Chronic kidney disease | 19 (17.6) | 3 (7.0) | 13 (18.8) | 35 (15.9) |
| Diabetes mellitus | 44 (40.7) | 6 (14.0) | 31 (44.9) | 81 (36.8) |
| General weakness/poor condition | 19 (17.6) | 6 (14.0) | 6 (8.7) | 31 (14.1) |
| Malignancy | 25 (23.1) | 24 (55.8) | 32 (46.4) | 81 (36.8) |
| Urinary catheterization | 14 (13.0) | 8 (18.6) | 15 (21.7) | 37 (16.8) |
| Prior therapies^a^ | 36 | 33 | 44 | 113 |
| Immunosuppressors | 10 (27.8) | 18 (54.5) | 30 (68.2) | 58 (51.3) |
| Antibiotics | 31 (86.1) | 28 (84.8) | 31 (70.5) | 90 (79.6) |

^a^During 3 months prior to IED onset.

*COPD* chronic obstructive pulmonary disease, *FAS* full analysis set, *IED* invasive *Escherichia coli* disease, *SD* standard deviation
