# Supplementary material for: Clinical presentation and antimicrobial resistance of invasive Escherichia coli disease in hospitalized older adults: a prospective multinational observational study
Source: Infection. 2024 Jan 25;52(3):1073–85. doi: 10.1007/s15010-023-02163-z (PMC11142950; doi:10.1007/s15010-023-02163-z)
Supplement: Supplementary file 9 — Supplementary file9 (DOCX 20 KB) [file 15010_2023_2163_MOESM9_ESM.docx]

**Table S8** Clinical manifestation of IED stratified by the infection acquisition setting (FAS)

|  | **Community-acquired** | **Hospital-acquired** | **Healthcare-associated** | **All IED** |
| --- | --- | --- | --- | --- |
| FAS^a^ | 121 | 48 | 71 | 240 |
| Sign, symptom, or clinical syndrome^b^, n (%) | 120 | 46 | 71 | 237 |
| Fever | 61 (50.8) | 29 (63.0) | 33 (46.5) | 123 (51.9) |
| Hypothermia | 10 (8.3) | 0 | 1 (1.4) | 11 (4.6) |
| Hyperleukocytosis | 58 (48.3) | 12 (26.1) | 32 (45.1) | 102 (43.0) |
| Leukopenia | 7 (5.8) | 14 (30.4) | 5 (7.0) | 26 (11.0) |
| Tachypnea | 28 (23.3) | 5 (10.9) | 25 (35.2) | 58 (24.5) |
| Tachycardia | 68 (56.7) | 29 (63.0) | 43 (60.6) | 140 (59.1) |
| General symptoms^c^ | 85 (70.8) | 23 (50.0) | 34 (47.9) | 142 (59.9) |
| Any laboratory values indicating bacterial infection and/or sepsis, n (%) | 99 (82.5) | 38 (82.6) | 58 (81.7) | 195 (82.3) |
| SIRS, n (%) | 110 (91.7) | 42 (91.3) | 66 (93.0) | 218 (92.0) |
| Sepsis, n (%) | 88 (73.3) | 36 (78.3) | 49 (69.0) | 173 (73.0) |
| Septic shock, n (%) | 14 (11.7) | 3 (6.5) | 7 (9.9) | 24 (10.1) |
| Any signs and/or symptoms of UTI, n (%) | 76 (63.3) | 16 (34.8) | 29 (40.8) | 121 (51.1) |
| ^a^Patient may appear in ≥1 category. ^b^Time of IED diagnosis. ^c^General symptoms include malaise, fatigue, muscle pain and chills.  *FAS* full analysis set, *IED* invasive *Escherichia coli* disease, *SIRS* systemic inflammatory response syndrome, *UTI* urinary tract infection. | | | | |
