# Supplementary material for: Clinical presentation and antimicrobial resistance of invasive Escherichia coli disease in hospitalized older adults: a prospective multinational observational study
Source: Infection. 2024 Jan 25;52(3):1073–85. doi: 10.1007/s15010-023-02163-z (PMC11142950; doi:10.1007/s15010-023-02163-z)
Supplement: Supplementary file 10 — Supplementary file10 (DOCX 18 KB) [file 15010_2023_2163_MOESM10_ESM.docx]

**Table S9** Antibiotic therapy used for the treatment of IED (FAS)

|  | **Bacteremic IED** | **Non-bacteremic IED** | **All IED** |
| --- | --- | --- | --- |
| Analysis set: FAS | 193 | 47 | 240 |
| Antibiotics prescribed^a^, n (%) |  |  |  |
| Ciproflaxacin | 92 (47.7) | 30 (63.8) | 122 (50.8) |
| Piperacillin-tazobactam | 57 (29.5) | 10 (21.3) | 67 (27.9) |
| Piperacillin | 35 (18.1) | 0 | 35 (14.6) |
| Amoxicillin-clavulanate | 27 (14.0) | 1 (2.1) | 28 (11.7) |
| Meropenem | 23 (11.9) | 5 (10.6) | 28 (11.7) |
| Vancomycin | 18 (9.3) | 6 (12.8) | 24 (10.0) |
| Ceftriaxone | 17 (8.8) | 4 (8.5) | 21 (8.8) |
| Amoxicillin | 15 (7.8) | 4 (8.5) | 19 (7.9) |
| Ampicillin | 15 (7.8) | 4 (8.5) | 19 (7.9) |
| Metronidazole | 15 (7.8) | 1 (2.1) | 16 (6.7) |
| Gentamicin | 11 (5.7) | 1 (2.1) | 12 (5.0) |

^a^Antibiotics used by ≥5% of all patients.

*FAS* full analysis set, *IED* invasive *Escherichia coli* disease.
