# Supplementary material for: Clinical presentation and antimicrobial resistance of invasive Escherichia coli disease in hospitalized older adults: a prospective multinational observational study
Source: Infection. 2024 Jan 25;52(3):1073–85. doi: 10.1007/s15010-023-02163-z (PMC11142950; doi:10.1007/s15010-023-02163-z)
Supplement: Supplementary file 11 — Supplementary file11 (DOCX 20 KB) [file 15010_2023_2163_MOESM11_ESM.docx]

**Table S10** Antibiotic resistance status among patients with IED stratified by mortality (FAS)

|  | **Mortality=Y^a^** | **Mortality=N^a^** |
| --- | --- | --- |
| Analysis set: FAS | 11 | 227 |
| Number of *E. coli* isolates with AST performed | 12 | 287 |
| Number of *E. coli* isolates resistant to the given antibiotic^b^ (%) |  |  |
| Amikacin | 1 (8.3) | 1 (0.3) |
| Ampicillin | 5 (41.7) | 163 (56.8) |
| Ampicillin/sulbactam | 0 | 56 (19.5) |
| Aztreonam | 2 (16.7) | 25 (8.7) |
| Cefazolin | 2 (16.7) | 54 (18.8) |
| Cefepime | 0 | 17 (5.9) |
| Ceftazidime | 0 | 13 (4.5) |
| Ceftriaxone | 2 (16.7) | 47 (16.4) |
| Ciprofloxacin | 4 (33.3) | 68 (23.7) |
| Colistin | 0 | 3 (1.0) |
| Gentamicin | 1 (8.3) | 32 (11.1) |
| Levofloxacin | 4 (33.3) | 62 (21.6) |
| Minocycline | 2 (16.7) | 16 (5.6) |
| Piperacillin/tazobactam | 0 | 5 (1.7) |
| Tetracycline | 5 (41.7) | 86 (30.0) |
| Tigecycline | 0 | 1 (0.3) |
| Tobramycin | 2 (16.7) | 28 (9.8) |
| Trimethoprim/sulfamethoxazole | 5 (41.7) | 86 (30.0) |
| Number of *E. coli* isolates resistant to ≥1 antibiotic in ≥1 drug class^c^ (%) | 7 (58.3) | 179 (62.4) |
| Number of *E. coli* isolates resistant to ≥1 antibiotic in ≥2 drug classes^c^ (%) | 6 (50.0) | 130 (45.3) |
| Number of *E. coli* isolates resistant to ≥1 antibiotic in ≥3 drug classes^c^ (%) | 4 (33.3) | 100 (34.8) |

^a^A patient may have more than one isolate test result. ^b^Denominator is total number of *E. coli* isolates with AST performed. ^c^Antibiotic drug classes: aminoglycoside, carbapenem, cephalosporin, fluoroquinolone, folate pathway inhibitor(s), nitrofurantoin, penicillin, penicillin/β-lactamase inhibitor, polymyxin/lipopeptide and tetracycline.

*AST* antimicrobial susceptibility test, *FAS* full analysis set, *IED* invasive *Escherichia coli* disease
