# Supplementary material for: Clinical presentation and antimicrobial resistance of invasive Escherichia coli disease in hospitalized older adults: a prospective multinational observational study
Source: Infection. 2024 Jan 25;52(3):1073–85. doi: 10.1007/s15010-023-02163-z (PMC11142950; doi:10.1007/s15010-023-02163-z)
Supplement: Supplementary file 12 — Supplementary file12 (DOCX 20 KB) [file 15010_2023_2163_MOESM12_ESM.docx]

**Table S11** Antibiotic resistance status among patients with IED stratified by the presence of sepsis (based on SOFA score) (FAS)

|  | **Sepsis=Y^ab^** | **Sepsis=N^ab^** |
| --- | --- | --- |
| Analysis set: FAS | 143 | 95 |
| Number of *E. coli* isolates with antimicrobial susceptibility test performed | 179 | 120 |
| Number of *E. coli* isolates resistant to the given antibiotic^c^ (%) |  |  |
| Amikacin | 1 (0.6) | 1 (0.8) |
| Ampicillin | 105 (58.7) | 63 (52.5) |
| Ampicillin/sulbactam | 36 (20.1) | 20 (16.7) |
| Aztreonam | 16 (8.9) | 11 (9.2) |
| Cefazolin | 31 (17.3) | 25 (20.8) |
| Cefepime | 10 (5.6) | 7 (5.8) |
| Ceftazidime | 6 (3.4) | 7 (5.8) |
| Ceftriaxone | 28 (15.6) | 21 (17.5) |
| Ciprofloxacin | 45 (25.1) | 27 (22.5) |
| Colistin | 1 (0.6) | 2 (1.7) |
| Gentamicin | 22 (12.3) | 11 (9.2) |
| Levofloxacin | 44 (24.6) | 22 (18.3) |
| Minocycline | 11 (6.1) | 7 (5.8) |
| Piperacillin/tazobactam | 2 (1.1) | 3 (2.5) |
| Tetracycline | 58 (32.4) | 33 (27.5) |
| Tigecycline | 1 (0.6) | 0 (0.0) |
| Tobramycin | 19 (10.6) | 11 (9.2) |
| Trimethoprim/sulfamethoxazole | 61 (34.1) | 30 (25.0) |
| Number of *E. coli* isolates resistant to ≥1 antibiotic in ≥1 drug class^d^ (%) | 113 (63.1) | 73 (60.8) |
| Number of *E. coli* isolates resistant to ≥1 antibiotic in ≥2 drug classes^d^ (%) | 83 (46.4) | 53 (44.2) |
| Number of *E. coli* isolates resistant to ≥1 antibiotic in ≥3 drug classes^d^ (%) | 68 (38.0) | 36 (30.0) |

^a^Sepsis=Y if the SOFA score is ≥2. ^b^A patient may have more than one isolate test result. ^c^Denominator is total number of *E. coli* isolates with AST performed. ^d^Antibiotic drug classes: aminoglycoside, carbapenem, cephalosporin, fluoroquinolone, folate pathway inhibitor(s), nitrofurantoin, penicillin, penicillin/β-lactamase inhibitor, polymyxin/lipopeptide and tetracycline. *AST* antimicrobial susceptibility test, *FAS* full analysis set, *IED* invasive *Escherichia coli* disease, *SOFA* Sequential Organ Failure Assessment.
